# Human control of AI systems: from supervision to teaming

**DOI:** 10.1007/s43681-024-00489-4

**Published:** 2024-05-28

**Authors:** Andreas Tsamados, Luciano Floridi, Mariarosaria Taddeo

**Affiliations:** 1 Oxford Internet Institute, University of Oxford, Oxford, UK; 2 Digital Ethics Center, Yale University, New Haven, USA; 3 Alan Turing Institute, London, UK

**Keywords:** Artificial intelligence, Foundation models, Human control, Human machine teaming, Cooperative AI, Supervisory control, Meaningful human control

## Abstract

This article reviews two main approaches to human control of AI systems: supervisory human control and human–machine teaming. It explores how each approach defines and guides the operational interplay between human behaviour and system behaviour to ensure that AI systems are effective throughout their deployment. Specifically, the article looks at how the two approaches differ in their conceptual and practical adequacy regarding the control of AI systems based on foundation models––i.e., models trained on vast datasets, exhibiting general capabilities, and producing non-deterministic behaviour. The article focuses on examples from the defence and security domain to highlight practical challenges in terms of human control of automation in general, and AI in particular, and concludes by arguing that approaches to human control are better served by an understanding of control as the product of collaborative agency in a multi-agent system rather than of exclusive human supervision.

## Introduction

Understanding how humans should––and do––control artificial intelligence (AI) systems is central to many research areas, with applications ranging from self-driving vehicles to cybersecurity and national defence. In this article, we focus on literature addressing the human control of automation in general, and AI in particular, within the defence and security domain. We analyse two main approaches to human control of AI described in the relevant literature, *supervisory human control* (SHC) and *human–machine teaming* (HMT) and assess their compatibility with the capabilities and operationalisation of AI systems based on foundation models. In doing so, our goal is to lay the groundwork for an approach to human control of AI systems based on foundation models.

Foundation models mark a significant shift in their capabilities and limitations compared to other types of AI, including rule-based AI, which have been examined in the extant literature on human control. The field of AI research recognises this category of models as an emerging architecture of AI adaptable to many downstream tasks [1]. Indeed, because they are pre-trained on vast datasets, generally by using self-supervision at scale, foundation models are uniquely versatile and easy to interact with: they can take multiple modalities of inputs (e.g., text, image, videos, code), as well as generate them, interact with the digital world via APIs and interact conversationally with human operators through natural language [1]. However, their scale and complexity lead to serious ethical and security challenges, including a lack of predictability and interpretability, model hallucinations (i.e., making up information), unwanted biases, and security vulnerabilities (e.g., prompt injections) [2]. These challenges create operational risks and can hamper our ability to use AI effectively in areas that would benefit from it. How to solve or at least mitigate them is the purpose of approaches to human control of AI systems.

In this article, we focus on the defence and security domain for two reasons. First, the relevant literature has debated issues of human control of AI for over a decade, especially since the 2012 US executive order on autonomy in weapon systems [3] and, before that, with extensive research on automation in air defence systems and unmanned vehicles during the second half of the twentieth century [4]. Research in this area provides many examples of real-world deployments, which help assess the validity of approaches to human control and their robustness across contexts [4, 5]. Second, this is a high-risk domain where lack of control can lead to material damage and harm, so acceptable solutions for controlling AI systems in this domain may be at least equally acceptable in other domains, such as healthcare or transportation, that are at most as risky.

**Fig. 1:**
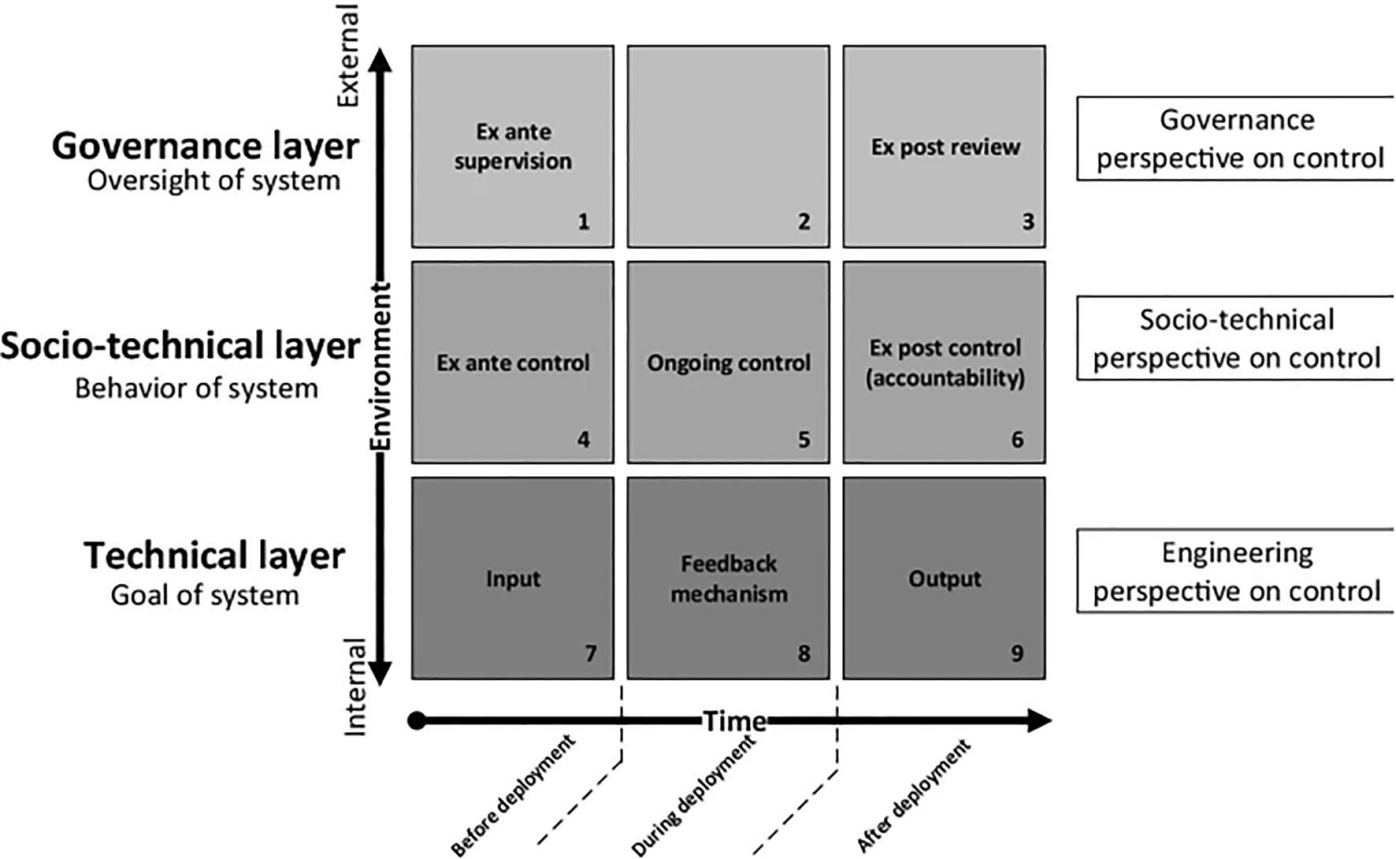
Comprehensive Human Oversight Framework [6, p. 151]

To help situate our work within the broader literature on human control of AI, we refer to Verdiesen et al.'s [6] Comprehensive Human Oversight Framework, illustrated in Fig. 1.

Figure 1 maps human control in terms of temporal phases (on the x-axis), namely before, during, and after deployment, and three environmental layers or perspectives, i.e. technical, socio-technical, and governance (on the y-axis). Our work focuses on the category of ongoing control (in box 5), which combines human and system control of AI systems during deployment. Ongoing control includes human behaviour, human decision-making, AI system behaviour, and their interplay during deployment. It focuses on the ability of the human operator to ensure that an AI system is effective during deployment and that human decision-making is applied in situations that require it. The need for human decision-making may be either pre-defined (e.g., a type of action by the AI that requires explicit human approval) or exceptionally required (e.g., an irregular environment or input for which the AI is not adapted, which leads to risks that call for a human operator’s intervention). To work, an approach to ongoing control should have a theoretical basis of control (i.e., an assessment of risks and how to address them) that matches with the operational reality of deployment (i.e., real-world data on the interplay of human and system behaviour across diverse contexts of deployment).^1^ Thus, an approach to ongoing control should establish a correct understanding of the type of behaviours expected from the agents (i.e. human operators and AIs) during deployment and make them supplementary to each other to minimize risks. In the rest of this article, we shall refer to ongoing control as operational human control, to stress that we refer specifically to the deployment phase and to human–machine (as opposed to machine-machine) control dynamics. Henceforth, operational human control is defined as the decision-making process and set of actions of one or more human operators ensuring that one or more AI systems are effective during deployment––that is, they are performant, safe, ethical, and legally compliant. SHC and HMT are two approaches to operational human control.

SHC, and to a lesser degree HMT, face conceptual and practical challenges when considering their applications to foundation models-based AI systems. Indeed, the capabilities and operationalisation of these models assume a different dynamic between agents during deployment than what has traditionally been understood in the literature on these approaches. The operational reality of these models’ deployment blurs the lines between environmental perspectives––particularly between the technical and socio-technical ones––and the temporal phases of control described in Fig. 1. For example, the conversational capabilities of these models have made prompt-based instructions the primary means of interaction between human operators and AI systems, hence requiring regular human interventions during deployment and with wide variety of possible inputs. In addition, human interventions also occur indirectly throughout a faster rotation of temporal phases as models are regularly updated with new human input, either through continual learning in online models, or through model fine-tuning by users, or reinforcement learning from human feedback (RLHF) and other regular updates that the providers of an AI system may push to their model or the system it is part of. This shift requires us to reconsider the validity of both SHC and HMT and determine if and how they can be adapted to ensure human control AI systems based on foundation models. We argue that foundational models, with advancements in cognitive modelling and human–computer interactions, have created an opportunity to move from approaches to control that confine human operators to degrees of supervision of specific tools, to configurations whereby artificial and human agents are parts of a collaborative agency that produces the desired state of control.

In the rest of this article, Sect. 2 analyses SHC as the predominant approach to human control that has been applied thus far to automation and AI systems that do not exhibit the characteristics of foundation model-based AI systems (henceforth traditional AI systems). This analysis provides an overview of the well-recognised operational challenges relating to SHC, such as the loss of situational awareness. Section 3 analyses SHC considering the growing operationalisation of AI systems based on foundation models, highlighting the (in)compatibility of SHC as an approach with the operational reality of such systems. Section 4 introduces the HMT approach and outlines reasons it is better placed to adapt to the operational reality of foundation models-based AI systems and address associated risks. Section 5 highlights four of the most relevant hindrances that must be overcome to adapt and implement this approach. Section 6 concludes our analysis.

## Supervisory human control and its challenges

The SHC approach assumes at least two interacting agents––a human and an artificial agent, where the human supervises the artificial agent. The supervisor’s role is typically broken down into five, time-sequential steps, as described in Table 1.

**Table 1:** Typical human roles presented as time-sequential steps, as described in [8, p. 740]

Step 1	**Planning** off-line what task to do and how to do it
Step 2	**Teaching** (or programming) the computer what was planned
Step 3	**Monitoring** the automatic action online to make sure that all is going as planned and to detect failures
Step 4	**Intervening**, which means the supervisor takes over control after the desired goal state has been reached satisfactorily or interrupts the automatic control in emergencies to specify a new goal state and reprogram a new procedure
Step 5	**Learning** from experience to do better in the future

The SHC approach focuses on allocating specific tasks between humans and artificial agents. The task allocation depends on the technical capabilities of the artificial agents. These are usually mapped with respect to its level of automation [9]. The Levels of Automation (LOA) taxonomy, introduced by Sheridan and Verplank [10], is a widely adopted and adapted classification to assess human control of artificial agents, and it continues to influence the way that organisations think about how advanced a given artificial agent is (Table 2) [10].

**Table 2:** Levels of Automation as described in [10, pp. 168–169]

Level 1	Human does the whole job up to the point of turning it over to the computer to implement
Level 2	Computer helps by determining the options
Level 3	Computer helps to determine options and suggests one, which human need not follow
Level 4	Computer selects an action, and human may or may not do it
Level 5	Computer selects an action and implements it if human approves
Level 6	Computer selects an action, informs human in plenty of time to stop it
Level 7	Computer does the whole job and informs human what it did
Level 8	Computer does the whole job and informs human what it did only if human explicitly asks
Level 9	Computer does the whole job and decides what the human should be told
Level 10	Computer does the whole job if it decides it should be done, and if so, informs human, if it decides that the human should be told

Sheridan and Verplank organise their taxonomy of automation into 10 (later reduced to eight) levels of automation. Finding and maintaining the adequate LOA is not trivial, since no proper guidance exists on how to apply and adapt the taxonomy to different contexts. Consequently, the relevance of this taxonomy––while still popular as a concept and despite its past use in defence organisations like the US Army––has waned even among its original authors [8, p. 743].^2^

However, the taxonomy helps consider an assumption underpinning the SHC approach, i.e., human–machine interactions are framed in analogy with how human supervisors interact with their subordinates [11]. According to Sheridan [8, pp. 736–737], in its strictest definition, SHC indicates that:

“…one or more human operators are setting intermittent subgoals to a computer, and receiving information from a computer, that itself closes an inner control loop through electromechanical actuators, the task, and feedback sensors. […] The human gives intermittent (typically symbolic) commands to the computer and receives intermittent feedback from the computer. The computer acts on the intermittent commands from the supervisor to close a continuous automatic control loop through the actuator, task, and sensors, while the human monitors.”

In practice, applying SHC and associated LOA taxonomies to specific applications unveils crucial limitations [8, 12]. The first one we identify refers to the loss of situational awareness of the human agent. Consider, for example, the US Patriot missile system, which has a history of committing so-called “friendly fire” and seeing “ghost targets”, which can be “partially attributed to a lack of understanding of human limitations in supervisory control” as well as a lack of procedures and standards to operationalise human control in the US Army [8, p. 743], [13]. In this case, the US Army admitted after an investigation that Patriot training standards were missing, “autonomous operations procedures” were unclear, and “operators commonly lose situational awareness of air tracks” [8, p. 743]. SHC and LOA, as formulated here, do not solve the overarching problems of effective communication and complementarity between human behaviour and system behaviour, nor do they address the cognitive load that human monitoring and intervention can exert on human operators, among other issues [15].

It is worth mentioning that multiple attempts have been made to codify SHC using different scales in LOA and/or changing the focus of the taxonomy from automation to the level of human input (e.g., Level of Human Control Abstraction) [16] or to the relationship between human and machine and the interpretability of their behaviours [17]. The LOA taxonomy has received extensive critiques in the process of being modified. For example, Endsley & Kaber [18] and Feigh and Pritchett [19] raise concerns about the lack of empirical validation and the overall utility of the taxonomy. Bradshaw et al. [20] raises questions about the exchange of roles between agents and the extent to which they can act as substitutes for each other. Kaber [21] points out that the taxonomy ignores the question of moral responsibility and changing LOA during operations [12, p. 743].

We agree with these criticisms and suggest that four challenges are relevant to understanding the extent of the limitations of the SHC approach. These are: the loss of situational awareness and the vicious cycle that it can engender, contextual changes that disrupt the established allocation of tasks among agents, issues of trust vis-à-vis a system, and bias towards controlling technical and performance aspects of the automated systems at the detriment of other dimensions of risk.

Loss of situational awareness is a recurring issue in the implementation of SHC [22]. Consider, for example, the Out-Of-The-Loop (OOTL) performance problem [9]. OOTL describes a situation where the human operator has low or no situational awareness and is slow in identifying a problem, or incapable of appropriately responding to it. OOTL has resulted in severe accidents in nuclear energy production and aviation, among other domains [21]. Most notably, the Three Miles Island nuclear incident [23] and the Flight Rio-Paris 447 crash [24], illustrate how human control factors contributed to catastrophic failures. In these cases, human operators were powerless to act when their systems failed as they could neither assess the cause of the failure nor understand how to resolve the situation in the absence of a working system or to fix the given systems [23, 24]. The complexity of the tasks that AI systems can undertake makes the problem of OOTL even more urgent. Bainbridge [25] calls this a classic challenge of automation or “ironies of automation”, whereby increased capabilities also increase the challenges faced by human operators [25]. The wider the capabilities of a system and its application surface, the lower the situational awareness of human operators and the less likely they are to be able to control the system effectively [9, p. 121].

The loss of situational awareness stems from a lack of systematic engagement by a human operator in each task or mission. This creates a vicious cycle whereby: operators’ skills deteriorate, they have reduced sensitivity to essential signals, and they develop complacent behaviours, which then lead to more cases of loss of situational awareness and the deterioration of trust vis-à-vis the system [9, 26]. This cycle is often exacerbated when because automated systems can be brittle in unforeseen contexts of deployment, creating challenges such as “automation surprise” for the human operator, who may have to intervene without having information about the state of the system and where it fell short operationally [21, p. 2].

At an organisational level, this vicious cycle culminates in higher levels of automation or more integrations of automated systems being considered as solutions to the human operator not being able to intervene effectively and efficiently enough [15, 27]. This cycle is a recurrent problem in human control of automated systems as new, unforeseen circumstances are always bound to happen during deployment phases and can undermine intervention processes prepared in advance for human operators [21, 28, 29]. However, it is also important to note that the likelihood of unforeseen circumstances occurring can be reduced by lowering the complexity of the deployment environment (e.g., limiting the environmental variables interacting with the system) and/or of the system itself. We return to this point in Sect. 3 as it highlights the difference in the technical complexity of traditional automation and foundation models.

The second limitation of SHC approaches appears when considering changes in the deployment environment. A minor change in the environment can lead to an inadequate context of operation for the system, in which case the human operator would need to understand (or be notified) that timely intervention is needed to prevent risks from being realised. This requires a more comprehensive understanding of human–machine interactions [30] than what is captured in the LOA taxonomy, which give way to system design and control interventions based on fixed task allocations among agents. To address this issue, Siebert et al. [31] propose to structure human control around a more extensive design space called the “moral operational design domain”, which builds on the concept of the operational design domain from the automotive industry. The purpose of this approach is to specify the contextual conditions that a given system ought to be operating in, and outside of which human intervention should be triggered, along with the moral responsibility of the involved operator. However, this approach is limited by our ability to specify contextual conditions in complex environments, as complex environments consist of many unknowns and evolving states. The complexity of both the automated system and the context of deployment (i.e. environment plus task at hand) will muddy the flow of information to the human operator with respect to the state of the system, environmental factors, the nature of the problems that have emerged and thus, the type of action that is required from the operator [32, 33]. When unforeseen situations develop, SHC lacks the flexibility required to dynamically re-allocate tasks or create a new set of interactions between the human operator and the AI system.

There is a tension in the relevant literature originating from the difficulty in defining the degree of human involvement across distinct types of automated systems or even the same systems in an evolving context. This tension is made more apparent by the advent of AI systems, which increases the complexity of automation and can imbue automated systems with capabilities pertaining to autonomy, such as self-governance, learning, and adaptability. Because of these changes, it is crucial to reconsider assumptions about human operators' role in supervisory control and the type of human–AI interactions that the literature should be designing for and optimising towards.

The third limitation concerns the level of trust that a human operator has vis-à-vis the AI systems, and it can stem directly from the vicious cycle that the loss of situational awareness can trigger. Human operators tend to over-trust and accept uncritically the outputs of AI systems, which induces operational complacency that is counterproductive to the SHC rationale [34, 35]. The longer a given AI system has performed well, the more likely a human operator is to trust it and perceive some kind of “mechanistic objectivity” associated with computer-generated analytics, for example [2, p. 212], [36]. This can lead to over-trust dynamics, whereby the human agents ignore their experienced assessments—so-called automation bias [37]—or even “shirking part of their responsibility for decisions”, when this would contradict the behaviour of the AI system.

The fourth limitation becomes evident when considering the specific types of risks that SHC aims to mitigate. These can be divided into performance and socio-legal control [12, 30]. They can be described as follows:

- Human control to mitigate and manage performance risks (aka performance control): human operators are considered capable of rectifying or replacing a given system if it malfunctions and/or becomes unreliable due to unexpected circumstances;
- Human control to mitigate and manage socio-legal risks (aka socio-legal control): the epistemic standing of human operators allows them to determine whether the context in which a system operates requires socio-legally-informed decisions, which the system is incapable of making. A related point is that such decisions require a morally accountable party, which cannot be the system itself.

Both categories need to be considered for an effective approach to human control of AI, albeit one may be prioritised in a specific context or when one category of risk is more likely or impactful than the other. However, existing approaches tend to focus on one at the detriment of the other [30]. This could be because, operationally, it can be problematic for human operators to mitigate both types of risks when intervening in a specific context. For example, an intervention of a socio-legal nature may require the human operator to stop the system's activity altogether to review the situation and address potential problems, in which case performance concerns are deliberately set aside. This has led to the framing of human–machine interactions and suggestions for human interventions that are biased towards performance considerations at the detriment of socio-legal ones, which are difficult to define practically (more on this presently) [30]. A specific type of SHC–*meaningful human control* (MHC)–emerged to address this bias in recent years. We delve into MHC in the next section.

### Meaningful human control

MHC has become a central topic of debate as it informs several policies and legal approaches concerning the control and deployment of autonomous weapon systems and lethal autonomous weapon systems. It focuses on moral responsibility, the conditions of meaningful control, and establishing an appropriate chain of accountability [38]. Despite being so central to the debate on the control of AI systems, a shared definition of MHC and research on operationalising it are still lacking [39]. Indeed, a standard categorisation used in association with MHC includes the three degrees of human control of autonomous weapon systems and lethal autonomous weapon systems [39, 40]:

(i) Human-in-the-Loop Weapons: “Robots that can select targets and deliver force only with a human command;
(ii) Human-on-the-Loop Weapons: Robots that can select targets and deliver force under the oversight of a human operator who can override the robots’ actions; and
(iii) Human-out-of-the-Loop Weapons: Robots that can select targets and deliver force without any human input or interaction” [39, p. 2].

These categories have been adopted widely, but it is worth stressing that they have yet to be paired with a comprehensive operational framework that details the practical aspects they each involve in different contexts of deployment.^3^

Recalling Fig. 1, MHC, ranges across the three deployment phases and in both the governance and socio-technical layers of the framework. Indeed, some of the literature on MHC advocates for the non-deployment (i.e., ex-ante control/oversight) of specific systems (i.e., lethal autonomous weapon systems) and the establishment of human-in-the-loop protocols (i.e., ongoing control) with a specific focus on chains of accountability following international humanitarian law (i.e., ex-post control/review). This spreads the focus of the MHC literature and makes it harder to derive regulations and practical guidelines from it––both for organisations deploying AI systems and human operators [41].

A key issue when considering MHC is determining appropriate levels of authority and moral responsibility. This is because protocols based on the MHC approach allocate tasks and authority without first defining a baseline for the conditions of deployment and how the change of conditions affects it. For example, they do not set thresholds for the abilities of the human operator (e.g. what level of technical understanding and training is necessary for MHC? What level of understanding about a specific system behaviour is sufficient to have MHC?) or for the level of robustness and predictability of the AI systems [31, 42]. In the human-factors literature [31, 43, 44], psychological and physiological principles have been applied to “support the identification of a realistic baseline on human ability” and the associated challenges that may emerge throughout human–machine interactions [31, p. 9]. However, existing approaches are limited when it comes to systems that regularly exhibit new and often unexpected capabilities and behaviours during deployment, as in the case of AI systems.

When considering automated systems, and especially AI systems, assessments are too often confined to benchmarks that measure system-specific metrics, like accuracy (e.g., in computer vision), which come at the detriment of assessing the entire systems’ behaviour and its relational capabilities vis-à-vis human operators [44, 45]. Designing appropriate human control approaches requires us to move beyond an atomic understanding of each agent’s capabilities to determine their appropriate level of authority and responsibility. This requires that the AI research community moves from benchmarking progress on whether AI systems can outperform humans in given tasks to assessing the performance of human–AI systems as a whole, including their interactive, organisational, and collaborative capabilities as agents of a more extensive system [46, 47]. For example, training programs that focus on testing human–machine interactions in realistic settings––either through high-fidelity simulations or deployment in contained environments––can reveal insights about agents’ capabilities and the corresponding levels of authority they should be given. Indeed, this is a growing trend among organisations spearheading the development of AI systems for complex socio-technical environments, such as the Defense Advanced Research Projects Agency (DARPA). The agency has launched several programs, including ASIST [48], SAIL-ON [49], and CAML [50] and associated software for assessing and improving human–AI systems’ performance during deployment or in computer-simulated environments, as well as to test AI systems in complex socio-technical environments (more on this in the next section).

Section 2 and the present one focused on autonomous artificial agents not endowed with capabilities exhibited by foundation models-based AI. We shall now analyse what specific challenges AI-enabled autonomy poses to the SHC approach.

## Challenges in applying SHC to foundation model-based AI systems

The limitations of the SHC approach described in the previous section indicate that this approach is not the best suited when considering more complex AI systems that can be prompted by human operators. One may argue that operational solutions could be implemented to overcome such limitations, such as, extensive training programs to pace the level of trust of the human agent in the artificial agents would mitigate issues emerging from the over-trust dynamics described in Sect. 2. However, solutions have yet to be established and SHC must be now considered for foundation model-based AI systems, which exacerbate some of the shortcomings of the approach with regards to human operators’ agency.

Technical factors challenge the use of the SHC approach to control foundation models-based AI systems effectively. Here, we analyse five of the most salient challenges and argue that effectively controlling these systems requires an alternative approach The first challenge emerges because foundation model-based AI systems are based on the scaling of “general purpose methods with increased computation and availability of large amounts of unstructured data” [2, p. 10], [51]. This means that the data processing done by AI systems occurs at a growing speed and scale that is cognitively prohibitive for human operators to supervise effectively, i.e. monitor, interpret, intervene on, and correct in a timely fashion. The second concerns the inability of foundation model-based AI systems to identify their limitations––that is, the capability to represent and communicate about tasks they cannot achieve [52]. Thus far, research has focused on a lower-level goal, namely, developing the ability of a system to identify when it cannot handle a situation and communicate it effectively. Note that this capability is also captured by the performance metrics that developers set for the AI systems, which can become too constraining for real-world environments or too permissive––meaning that constant re-evaluation is crucial to the successful implementation of these capabilities. This is essential to helping human operators correctly identify when and where their intervention may be required and how to reallocate tasks. The DARPA CAML program is a good example of ongoing research efforts to address this problem, as it is designed to improve AI systems’ capabilities to

“communicate their task strategies, the completeness of their training relative to a given task, the factors that may influence their actions, or their likelihood to succeed under specific conditions” [50, p. 1].

The third challenge stems from the non-deterministic behaviour of foundation model-based AI systems, making it challenging for human operators to know what behaviour to expect from the AI system and, thus, what anomalous behaviour would look like [2, 53]. Approximations and general expectations can be set by evaluating the AI system’s sensitivity to data, re-evaluating when new data is introduced and predicting the potential changes they would have on the system’s behaviour. However, the problem of predictability of AI systems remains and will often grow in parallel to the complexity of the environment and task at hand [2]. Foundation models have a quasi-boundless space of outputs and behaviour due to them being trained on web-scale datasets and the models comprising tens of billions of parameters; hence, the space of possible behaviours is also multiplied by the number of variables in the context of deployment. A related issue is that foundation models make it difficult to determine their actual capabilities as opposed to what capabilities they appear to exhibit in well-defined tasks. This lack of certainty over AI systems’ capabilities and, by extension, their range of behaviours, makes it difficult to pre-define a set of human interventions and establish when human intervention is required. Without symbolic reasoning or a model of causal relations to understand, for example, language, this architecture has led to models exhibiting unexpected and occasionally unwanted behaviour, such as presenting fictitious information as facts [13] or being sensitive to adversarial examples [2]. This is crucial from the point of view of human control because it is unclear how to address these limitations. From a technical perspective, developers have sought to align their models with human values to solve this unexpected, unwanted behaviour issue through a fine-tuning process based on targeted human feedback and labelling called RLHF. This process involves creating new data based on the human evaluation of a model’s output to train a reward model favouring outputs aligned with human preferences. However, this approach has yet to be proven scalable. It assumes that reward model generalisation will always occur and be sufficient for downstream applications, yet the process is costly and slow as it relies on outsourced human labour sifting through ever-growing swaths of data [54]. From a socio-technical or operational level, the capabilities of foundation models-based AI rest heavily on human operators’ ability to prompt, verify, and correct outputs [55]. Autoregressive large language models––such as ChatGPT––especially, have enabled numerous new applications where the exhibited model capabilities depend often on an external system or an agent’s presence and ability to verify outputs and try new inputs to extract a desired output and mitigate hallucinations.^4^ In other words, it is challenging to use SHC to reconcile the fact that the level of automation has increased in terms of AI system capabilities and general applicability, while the system has also become much more dependent on human input at all levels (e.g., from regular prompting and the need for verification of each output, to alignment efforts via RLHF).

The fourth challenge follows from the growing complexity of these systems and the tasks they fulfil, which makes it challenging to create human–machine interfaces that reveal enough relevant information about the AI systems’ operations, state, and problems encountered without overwhelming the operator with information and options to act. Indeed, both humans and AI agents within a human–AI configuration ought to have some form of “representations of the involved tasks, role distributions, desired outcomes […] mutual capabilities” [31, p. 6]. This fosters effective collaboration, adaptability to new situations, and trust. However, the more complex the foundation models-based AI system’s behaviour and the wider its application surface is, the harder it is to design interfaces that capture the state of the model, levels of uncertainty for each output, and other context-specific information needed for human operators to construct an accurate mental representation of the AI and assess the need for intervention [14, 56]. The appropriate interface is difficult to design as the path from input to output depends on a prohibitive scale of parameters.

SHC implies putting human operators in a crucial role with the agency over every tool or agent contributing to fulfilling a task. However, when considering foundation model-based AI systems, human operators are stripped of their operational agency in ways that contradict the purpose of human control. Moretto et al. showed that task motivation is increased when the participant can establish control over an effect, whereas the “loss of agency has been also proved to disturb the attribution of [moral] responsibility” [57, p. 5]. This leads to the fifth challenge, which refers to the impact of the increased autonomy of foundation models-based AI systems and the (loss of) sense agency among human operators, which, in turn, has induced a moral disengagement regarding the actions and decisions taken [58]. This is a dangerous consequence that is detrimental to human control for socio-legal purposes.

If the abilities of human operators diminish due to the plethora of tasks fulfilled by foundation model-based AI systems, both the authority they retain over a system and the responsibility they ought to bear for its behaviour should reflect that. Indeed, research has found a “decrease in agency concomitant with the increase in automation” [9, p. 121]. The introduction of foundation model-based AI systems removes human operators from action outcomes and “decreases their sense of control” and overall performance [9, p. 121]. Research on error-related potentials, or “cerebral activity associated with the monitoring of the consequences of an action” has shown a “degradation of monitoring associated with a reduction in the sense of agency” [6, p. 120]. The SHC approach does not capture this change even in its reviewed formulations. While it is still too early to tell, the issue of loss of sense of control can be expected to grow due to the versatility exhibited by foundation models on a range of tasks and the lack of measure of information accuracy or confidence intervals available to human operators [22, 59]. This creates a risk of exacerbating operators’ dependence on the system’s outputs and encourages epistemic vices, like the automation bias mentioned above [2], voiding any expectation of effective control of foundation model-based AI systems.

The limitations and the challenges to the SHC approach described in Sect. 2 and the present section highlight that the more the capabilities of AI systems grow, the less the supervisory role of the human agents is adequate to control such capabilities effectively. This is not just because technical aspects of AI systems hinder the implementation of the SHC approach, but also because the SHC approach focuses only on interventions to correct the system when something goes wrong; we refer to it as negative control. This is sufficient when considering lower levels of autonomy or even traditional AI systems. However, foundation model-based AI systems exhibit new capabilities, and leveraging these capabilities requires a different form of control. This enables a deeper integration between human behaviour and system behaviour and allows the human agents to contribute more consistently to the behaviour of the system, and leverage its capabilities to perform tasks but also ensure control. We refer to this as positive control. This has led us to consider the literature on HMT and cooperative AI to capture the collaborative dynamic of human–AI systems or teams and think about the operationalisation of human control. In the next section, we delve into the HTM approach and its implications for controlling foundation model-based AI.

## The human–machine teaming approach to foundation model-based AI systems

Research on HMT looks at integrating artificial agents into teams not as tools but as agents with autonomy, adaptability, and collaborative capabilities to create a larger, multi-agent system more capable than individual agents or agents interacting without a guiding framework [60]. However, human control is not a central element of research on HMT, which focuses more on performance-driven considerations––i.e., how a task can be achieved better through combining human abilities and system capabilities [60].

HMT has been described differently across the extant literature. O’Neil and McNeese [60, p. 2] explain that HMT involves:

“at least one human working cooperatively with at least one autonomous agent, where an autonomous agent is a computer entity with a partial or high degree of self-governance with respect to decision-making, adaptation, and communication”.

Wynne and Lyons have focused on the notion of teaming or partnering with a system, and on describing what makes the agent ascend to the status of an autonomous agent instead of simply a tool [61]. That is an important focus; sometimes, HMT is misused to describe human interactions with machines lacking the ability to self-govern, learn, or adapt [62]. Madni and Madni [32, p. 2] described HMT as being inherently adaptive and requiring:

“transparency in machine operations, bi-directional human–machine interaction, contextual awareness to understand changes in priorities and performance conditions, the ability for the human to intervene at different levels in ongoing machine processes to redirect resources, revise goals, and add or delete constraints.”

To avoid a common confusion in the extant literature, whereby kinds of human–machine interactions–even a simple one–might be referred to as HMT, in the rest of this article, we will use HMT only to describe configurations of collaborative agency in which task allocation and role assignment is dynamic and adaptive to the self-governance of the agents, rather than deterministic and pre-conditioned, and in which the output of the team stems from the collaboration among the agents. This requires that the artificial agent in HMT reaches a certain degree of autonomy that distinguishes it from automated systems as they are traditionally understood.^5^ Foundation model-based AI systems exhibit such autonomy.

The framing provided by the HMT approach comes with four requirements for establishing human control of AI systems. We base this list on a modified version of Dafoe et al.’s [46] work on cooperative AI, informed by common factors and challenges reported in the HMT literature. First, HMT research has focused on resolving the lack of shared representations or mental models (i.e. shared knowledge and understanding) between agents of a team on the tasks they share, the roles of each agent, their known capabilities and limitations, the influence of the environment in which they operate and the boundaries within which they should predictably operate [63, 64]. This is because shared representation of the system at large and of the operational environment allows agents to establish an action space with higher certainty and reduced risks as they can expect behaviours within this space, and, more importantly, understand when a given behaviour has wholly deviated from it. In high-stakes environments where changes are constant, and adaptivity is crucial, shared representation can help maintain a common ground around which agents can re-organise [65, 66]. Shared representation and alignment among agents are achieved through a combination of factors, including the quality of human–machine interfaces [67, 68]; convention building among teammates [2, 69]; and repeated team training exercises [65]. This is arguably the most complicated requirement when it comes to foundation models due to their limitations, including, (un)interpretable internal world models, lack of reasoning and planning capabilities and (un)predictability [17, 70, 71].

The second requirement is effective communication channels and feedback loops between agents [72]. The changes in internal states for the artificial agents (e.g., data sensitivity or malfunction) and human agents (e.g., cognitive, or emotional) need to be communicated adequately during operations to enable agents to change their expectations about their teammates and/or step-in when needed [72]. Communication in collaborative tasks can also indicate degrees of confidence or preference when faced with multiple options, expressing intended actions, or providing notice for imminent action concerning a given task [73]. Communication is also crucial in sharing newly discovered information and propagating it to the rest of the system so other agents can learn. For example, discovering environmental affordances in complex environments is a continual process that agents can share. Communication of malfunction or context-specific limitations or attacks are also key features of communication in HMT that would allow agents to update their understanding of one another’s capabilities and thus modify levels of authority accordingly.

The third requirement refers to commitment––in the sense of locking in an action or behaviour––is important in HMT as far as it increases the predictability of teammates’ actions where needed and can be requested by agents in cases of concerted, focused efforts where too many behavioural changes would be detrimental. This is advantageous for human control and risk management as it can aid in identifying deviance from committed behaviour, revealing potential malfunction or attack against an agent [74]. Commitment problems are ubiquitous in human teams and well documented as being detrimental to team composition and integrity [46, 75, 76].

The fourth requirement refers to building conventions and norms. This is key for well-functioning teams, whether HMT or fully human teams [77, 78]. Shared beliefs and understanding are built through pre-established parameters or can emerge naturally as the agents have recurrent interactions over a long time. Conventions lead to developing team-specific expectations, unspoken rules and even language [62, 73]. Conventions and norms building are derived from, and improve, understanding, communication, and commitment [64]. Regarding human control, conventions and norm building enabled by HMT create more familiar and predictable behaviours. This can lead to shorter communication times and more timely interventions [64]. It can also help specify Siebert et al.’s [31] moral operational design domain of artificial agents, such that after many different deployments, the team will have identified a set of recurrent contextual conditions that should automatically constrain specific agents' behaviour. It is also worth emphasising here that conventions and norms building also breeds trust in HMT [61, 69, 79]. A well-established team with internal conventions and experience being deployed as a unit would be less likely to fall into the trap of over-trust and complacent attitudes or develop distrustful interactions due to the absence of shared expectations [2].

## HMT for real-world deployment of foundation models-based multi-agent systems

These four requirements in HMT highlight important factors concerning human–AI interactions that can contribute to formulating a novel approach for controlling foundation model-based AI systems. However, understanding the extent to which these requirements should be met to create a robust human–AI configuration for control requires extensive testing. Testing HMT in real-world settings, evaluating different approaches to the operationalisation of HMT, and obtaining enough empirical data to deploy such systems with assurance has proven difficult [65, 77]. This acknowledgement of the need for more testing and for “exploring the uncertainty” inherent in HMT in complex environments without assuming high levels of risks has been a familiar rallying cry among defence and security organisations across the world [65, 79–81] and in other domains of application such as in healthcare [82]. In a recent systematic review, O’Neill et al. analysed 76 empirical studies of HMT. They found a severe lack of testing and evaluation in real-world settings, reporting that the current research on HMTs has been primarily

“conducted in laboratory environments involving simulation-based command and control, emergency rescue, and other computer games that require cooperation and communication among team members to complete tasks (e.g., B4WT [blocks for world teams])” [77, p. 6].

Indeed, examples of such confined testing environments abound. The USARSims is a search-and-rescue game in which a multi-agent team explores an unknown environment and identifies as many positions of “victims” as possible [78]. Another example is the Cognitive Engineering Research on Team Tasks–Unmanned Aerial System–Synthetic Task Environment (CERTT- UAS-STE) that is based on the US Air Force Predator UAS ground control station and requires “three interdependent teammates in distinct roles (pilot, navigator, and photographer) to take photographs of waypoints” [83, 84]. Moreover, most empirical studies of HMTs focus on simulated team tasks over a short time [60, 84]. This leads to a paucity of real-world data and longitudinal studies investigating long-term team development in real-world contexts and the utility of HMT factors in improving human control of AI systems. Thus, this gap must be closed to develop and use an HMT-based approach to control foundational models in high-risk domains, like the defence and security domain.

As Madni and Madni [32] suggest, the testing of joint human–AI performance in various operational contexts can also be simulated to accelerate HMT experimentations and inform practical guidelines for a generalisable approach to human control that moves beyond the constraints of supervisory human control of AI. However, evaluating HMT configurations requires holistic experiments, which are cost-intensive in the case of non-deterministic systems that generate consistently new and unexpected outputs. Both the pre-training and fine-tuning of foundation models, as well as the high-fidelity simulation of HMT scenarios, will require high levels of compute in many cases, making HMT research inaccessible to many researchers [67].

The spread of autoregressive large language models across consumer applications and the establishment of foundation models across domains also create an opportunity to contribute to closing the testing gap, to the extent that researchers can observe and test the performance of human–AI configurations in new contexts and under different levels of risk and benefit. Research focused on translating the general gain in AI systems' capabilities to improve human operators' ability to interact with and leverage AI will be critical to unlocking the potential of HMT-based approaches to human control. As foundation models seem poised to become an essential, long-term, and potentially problematic addition to humanity’s technological arsenal, making early research investments into developing a generalisable approach to human control of AI is essential.

Another gap to close concerns the absence of clear variables that can help us identify and assess socio-legally compliant behaviour, as reported by O’Neil et al. This is an obstacle to the HMT approach, which serves as the primary guiding approach for human control. However, rather than an obstacle caused by a fundamental incompatibility of HMT with socio-legal compliance, this reflects a research gap and an opportunity for further study. Indeed, rather than just seeking to combine human abilities and AI capabilities to complete a task more efficiently, the HMT literature can benefit from expanding its research into how this combination also creates a more robust entity vis-à-vis unforeseen risks of all types, including unprecedented socio-legal risks.

Preliminary to the development of any approach to control AI systems, whether based on foundation models or not, is the identification of acceptable risk thresholds. To this end, it is crucial to conduct a systematic risk analysis of the deployment context and consider the extent to which AI can cause damage and harm and the risk appetite linked to specific uses of AI technologies. Indeed, it may be that our risk tolerance for a particular task or environment is too low and incompatible with the unpredictable behaviour that is inherent in AI systems (thus far) and that even with human control, the integration of AI would not become a net benefit from an operational perspective. The reverse is also true: an AI system can be unreliable but still considered controlled insofar as its specific context of deployment may value the operator’s ability to leverage, for example, hallucinations for creative tasks, more than predictability. The decision-makers will assess risk appetite from time to time. However, standards must be developed to assess the type and level of risks that specific technologies may pose.

## Conclusion

This review has shown that the SHC approach to AI systems suffers from a series of limitations accentuated by the development and adoption of AI systems that rely, in whole or in part, on foundation models. This is because the autonomy that AI confers to artificial agents is directly at odds with the assumptions of supervisory human control. Having reviewed the literature on HMT, we argue that HMT offers a better framework to develop an alternative approach to human control of foundation model-based AI systems that focuses on bi-directional interactions and can be generalised to different areas of AI application.

The hypothesis that HMT can offer a productive path forward for the human control of AI systems rests on the assumption that the pace of innovation in AI research remains centred on foundation models instead of data and compute-efficient architectures that produce more explainable and predictable AI systems (e.g., neuro-symbolic AI). In the event of a departure from the non-deterministic and inscrutable models, we described in this analysis, supervisory human control may re-emerge as the primary approach to controlling AI systems. Nevertheless, as our analysis contends, the benefit of exploring HMT remains useful and transferrable to future human–AI configurations as it explores AI systems' cooperative and collaborative capabilities, the expectations of human operators, and the characteristics of an effective multi-agent system. Research investment into HMT and collaborative agency can be beneficial in the long term, irrespective of a change in AI capabilities, as it focuses on approaches that improve the quality of human–AI interactions and co-action.

## Footnotes

1 It is also worth mentioning that the absence of a named category in Box 2. This highlights a gap in the governance of control at the time of the publication of Verdiesen et al. [6]. However, today, promising methodologies have emerged to address this gap––consider for example continuous auditing of AI (CAAI) [7].

2 By Sheridan’s own admission [8], this was a rudimentary, qualitative idea that was not intended as a guideline, but was instead meant only to provide a way to think about machine behaviour and human roles. Nonetheless, SHC has been adopted and adapted in many areas since then, including home automation, self-driving cars, aviation, space exploration, manufacturing, hospital operating rooms, and for all kinds of robots used in different types of activities [8, p. 736], [14].

3 These three degrees of control are also used beyond MHC research, such as in machine learning (ML) research, but describe processes that are operationally different from the above descriptions. For example, Human-in-the-Loop has been used in ML research in recent years to describe a process whereby human feedback is used to improve the quality of a particular prediction model [41]. Human-in-the-Loop is used in the “Safety Best Practice” of the AI assistants ChatGPT, defined as the process of having human review model outputs, especially in “high-stakes domains”[42]. This contributes to the fragmentation of the discussion on control as different disciplines and application domains adopt similar terminology to describe operationally disparate control processes.

4 Note that a majority of mitigations to hallucinations have been dependent on rule-based verifiers and retrieval augmentation [56]

5 Note that we refer to a higher degree of autonomy here in a general sense and do not imply that autonomy ought to or even can be measured on a one-dimensional scale as it is context dependent and can fluctuate, among other reasons.
